# Syllabus of Obstetrical Lectures

**Published:** 1892-01

**Authors:** 


SYLLABUS OF OBSTETRICAL LECTURES.—By Richard C. Norris, A. M., M. D., Demonstrator of Obstetrics University of Pennsylvania, Physician to the Methodist Episcopal Hospital. Published by W. B. Saunders, Philadelphia, Pa.

A second edition being published as soon as sufficient evidence that such works are in demand.

While such compends should not take the place of text books, yet there is much in this work to commend.

It is brief, concise, tersely written, and contains many points of interest for the general practitioner as well as student.

It is rare to find so many good things in so small a work.

The blank leaves add to its usefulness for inserting individual opinions.

As a compend it will certainly prove useful.

